# Allosteric modulation of protein-protein interactions by individual lipid binding events

**DOI:** 10.1038/s41467-017-02397-0

**Published:** 2017-12-19

**Authors:** Xiao Cong, Yang Liu, Wen Liu, Xiaowen Liang, Arthur Laganowsky

**Affiliations:** 1 Institute of Biosciences and Technology, Texas A&M Health Science Center, Houston, TX 77030 USA; 2 Department of Chemistry, Texas A&M University, College Station, TX 77843 USA; 3 Present Address: Wolfe Laboratories Inc., 19 Presidential Way, Woburn, MA 01801 USA

## Abstract

The diverse lipid environment of the biological membrane can modulate the structure and function of membrane proteins. However, little is known about the role that lipids play in modulating protein–protein interactions. Here we employed native mass spectrometry (MS) to determine how individual lipid-binding events to the ammonia channel (AmtB) modulate its interaction with the regulatory protein, GlnK. The thermodynamic signature of AmtB–GlnK in the absence of lipids indicates conformational dynamics. A small number of lipids bound to AmtB is sufficient to modulate the interaction with GlnK, and lipids with different headgroups display a range of allosteric modulation. We also find that lipid chain length and stereochemistry can affect the degree of allosteric modulation, indicating an unforeseen selectivity of membrane proteins toward the chemistry of lipid tails. These results demonstrate that individual lipid-binding events can allosterically modulate the interactions of integral membrane and soluble proteins.

## Abstract (web summary)

Native mass spectrometry (MS) is a technique that preserves non-covalent interactions in the mass spectrometer. Here the authors use native MS to study integral membrane proteins, and find that lipids with different headgroups and tails can allosterically modulate protein-protein interactions in different fashions.

## Introduction

The regulated transport of ions and solutes across biological membranes is an essential chemical process. Regulation of ion channels is carried out by their molecular interactions with regulatory proteins, small molecules, lipids, or through post-translational modification^1–4^. Although some channels have been shown to be regulated by specific lipid–protein interactions, such as potassium channels by phosphoinositides^5^, beyond these specific examples we have very limited knowledge of how the chemically diverse lipid environment of the biological membrane modulates the structure and function of membrane protein.

As a step toward studying membrane protein and soluble protein interactions, we selected for study the ammonium channel (AmtB) from *Escherichia coli* (*E. coli*), an integral membrane protein that is regulated by the soluble trimeric protein GlnK^6, 7^. The interaction between AmtB and GlnK is tightly controlled by cellular nitrogen status through key effector molecules, while adenosine diphosphate (ADP) alone is sufficient to form the complex^8–10^. Atomic structures have revealed that GlnK blocks ammonia transport by essentially plugging the conducting channel within each subunit primarily through the insertion of a long surface loop (Supplementary Fig. 1)^11, 12^. Importantly, no lipids are resolved in the crystal structures of the AmtB–GlnK complex. Along these lines, there are 19 additional atomic structures of AmtB deposited in the Protein Data Bank^13^. For one of the crystal structures of AmtB, native mass spectrometry (MS) was used to identify a lipid that stabilized the channel, which guided co-crystallization trails leading to the first structure of AmtB bound to lipid^14^. However, for the other structures no bound lipids were observed, implying that crystallography will not always be adequate in the identification and biophysical characterization of lipids involved in membrane–protein interactions, emphasizing the necessity for new techniques to investigate protein–lipid interactions.

To determine how individual lipid-binding events modulate the interaction of AmtB with GlnK, we employed native MS, a powerful biophysical technique that has emerged over the past two decades to study proteins and their interactions with ligands^15–20^. Unlike other biophysical techniques, which are often ensemble measurements, native MS can preserve non-covalent interactions in the gas-phase as well as resolve and interrogate individual ligand-binding events to protein complexes^19–22^. Advances in native MS spanning nearly a decade^23^ have led to the ability to study membrane protein complexes in a native-like state that can provide invaluable information on their interactions with ligands, such as nucleotides, drugs, peptides, and lipids, and subunit stoichiometry^14, 24–29^. Recently, native MS coupled with a temperature-controlled source has been applied to monitor thermal unfolding^30^ as well as determine thermodynamics for protein–ligand interactions, including membrane protein–lipid interactions^31^. Importantly, thermodynamic parameters for soluble protein–ligand interactions determined using native MS are in agreement with those obtained using other biophysical techniques, such as isothermal titration calorimetry and surface plasmon resonance (SPR)^31, 32^. Building upon these recent advances, we use native MS to reveal that individual lipid-binding events allosterically modulate integral membrane protein and soluble protein interactions.

## Results

### Biophysical characterization of the AmtB–GlnK complex

**Fig. 1:**
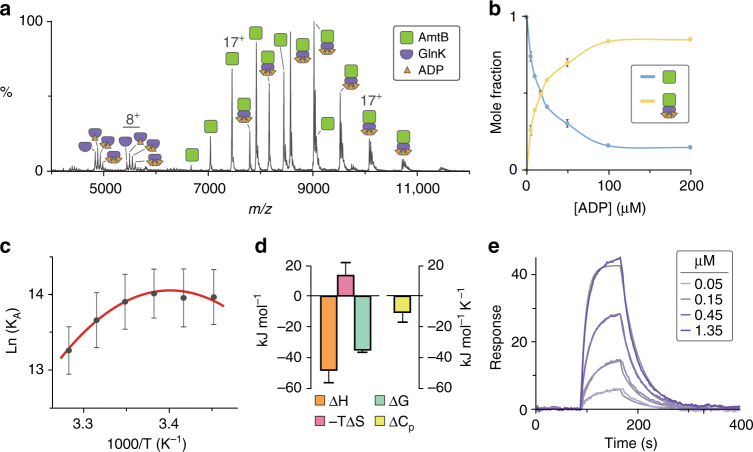
Biophysical characterization of the AmtB–GlnK complex by native MS and surface plasmon resonance. **a** Native mass spectrum of AmtB–GlnK at 2 μM in the presence of 50 µM ADP. **b** The effect of ADP concentration on GlnK association with AmtB determined by native MS. Plot of the mole fraction of AmtB and AmtB–GlnK as a function of total ADP concentration. Reported are the average and s.e.m. from repeated measurements (*n* = 2). **c** van’t Hoff plot for AmtB–GlnK binding in buffer containing 50 µM ADP (gray dots) as determined by native mass spectrometry and resulting fit (*R* ^*2*^ = 0.98) of the nonlinear “van’t” Hoff equation (red line). Reported are the average and s.e.m. from repeated measurements (*n* = 4). **d** Thermodynamic signature of AmtB–GlnK binding derived from native MS in the absence of lipid at 298 K. Reported are the average and s.e.m. from repeated measurements (*n* = 4). The change in free energy (Δ*G*) was calculated directly from repeated measurements of *K*_D,AG_ at 298 K, and entropy (Δ*S*) was back calculated using both Δ*H* and Δ*G* (Supplementary Table 1). **e** Representative sensorgrams of AmtB injected at different concentrations over a sensor surface immobilized with GlnK at 298 K (purple lines) and resulting fit of a Langmuir 1:1 binding model (gray lines). Reported are the average and s.e.m. from repeated measurements (*n* = 4)

We recorded a mass spectrum for the AmtB–GlnK complex in ammonium acetate buffer containing 50 μM ADP and two times the critical micelle concentration of tetraethylene glycol detergent (C_8_E_4_), a detergent that exhibits charge reducing properties^33^, and at a set temperature of 298 K using a temperature-controlled source^31^. The mass spectrum revealed an equilibrium of GlnK, AmtB, and AmtB–GlnK (Fig. 1a). Around 5000 *m/z*, we observed signals corresponding to apo and ADP_1–3_ bound states of GlnK. The mole fraction of GlnK(ADP)_0–3_ in the mass spectrum is in agreement with those calculated using reported equilibrium dissociation constants (*K*_D_) for GlnK-binding ADP_1–3_ in the absence of detergent (Supplementary Fig. 2)^31^. In addition, the measured mass for AmtB–GlnK is in agreement with three molecules of ADP bound. However, under our experimental conditions, we did observe a small signal corresponding to an additional ADP bound to both AmtB–GlnK and AmtB. This is likely non-specific adduction due to the high concentration of ADP and dissipates with lower ADP concentrations. These non-specific adducted peaks were taken into account when calculating the mole fraction of AmtB and AmtB–GlnK, and we determined the equilibrium dissociation constant for GlnK-binding AmtB (*K*_D,AG_) to be 1.12 ± 0.41 μM in the presence of 50 μM ADP.

To investigate further the influence of ADP concentration on GlnK binding to AmtB, we recorded mass spectra for a titration series of ADP and determined *K*_D,AG_ in a similar fashion described in the preceding paragraph (Fig. 1b and Supplementary Fig. 3). The *K*_D,AG_ varied from 3.8 μM at the lowest ADP concentration (5 μM), whereas at the highest concentration of ADP (200 μM) the equilibrium-binding constant dropped to 77.68 nM. At and above an ADP concentration of 100 μM, the *K*_D,AG_ approaches an asymptote around ~80 nM (Supplementary Fig. 3). From these experiments, a concentration of 50 μM ADP was selected to give an equilibrium of AmtB and AmtB–GlnK in approximate equal abundance, which is ideal for probing the effects of temperature and lipid binding (discussed below) on the AmtB–GlnK equilibrium-binding constant and was thus used in the following studies.

To determine the molecular forces behind the molecular recognition of GlnK by AmtB, we recorded mass spectra at a fixed ADP concentration (50 μM) and different temperatures. We then performed a ““van’t”” Hoff analysis^34^ to extract the thermodynamics for the AmtB and GlnK interaction. A plot of the natural log of *K*_D,AG_ as a function of the reciprocal of temperature was initially analyzed by fitting the “van’t” Hoff equation^34^ to the data, which resulted in a poor fit (*R* ^*2*^ = 0.68). In contrast, using the nonlinear form of “van’t” Hoff equation^35^ yielded exceptional fits with an *R* ^*2*^ of 0.98, indicating that heat capacity is not constant over the selected temperature range (for review see ref. ^36^). Using the nonlinear van't Hoff equation enabled us to determine the change in heat capacity (Δ*C*_p_) and change in enthalpy (Δ*H*) at a reference temperature of 298 K (Fig. 1c, d). The change in free energy (Δ*G*) was calculated directly from repeated measurements of *K*_D,AG_ at 298 K, and entropy (Δ*S*) was back calculated using both Δ*H* and Δ*G* (Supplementary Table 1). In short, the nonlinearity in the “van’t” Hoff plot suggests significant temperature-dependent conformational changes^36^ in AmtB and/or GlnK.

To corroborate our findings by native MS, we used SPR with GlnK immobilized on the sensor surface. Sensorgrams for different concentrations of AmtB were recorded at different temperatures in the same buffer and detergent used for native MS studies (Fig. 1e and Supplementary Fig. 4). Fitting the data to a Langmuir 1:1 binding model resulted in a *k*_on_ of 5.49 × 10^4^ M^−1^ s^−1^ and *k*_off_ of 3.14 × 10^–2^ s^−1^ (Supplementary Table 1). The average *K*_D,AG_ was determined to be 0.65 µM, which is in accord with that measured by native MS. We also observed similar nonlinear “van’t” Hoff plots for measurements obtained using SPR (Supplementary Fig. 4). Performing a similar analysis used for native MS data, we obtained similar thermodynamic parameters from SPR measurements substantiating our native MS findings (Supplementary Table 1).

Thermodynamic parameters elucidated by native MS and SPR provide additional insight into the molecular driving forces that drive association of AmtB and GlnK. The AmtB–GlnK interaction is largely driven by enthalpy, which is consistent with the crystal structures of the complex^11, 12^ where a total of 39 hydrogen bonds, six of which are mediated through water bridges, are formed between the trimeric assemblies (Supplementary Fig. 1). In addition, there is a large interface area of 2704.8 Å^2^ formed between AmtB and GlnK (PDB 2NS1)^37^. The large negative Δ*C*_p_ yields an upward curvature to Δ*G* with lower temperature ranges having smaller *K*_D,AG_ values. In addition, this Δ*C*_p_ value also suggests that these macromolecules adopt a flexible conformation or a variety of interconverting conformations^38^. Different conformations are supported by crystallographic structures, where a conformational change in AmtB is observed when bound to GlnK that arises from rigid body movements of several transmembrane helices within each subunit (Supplementary Fig. 1c)^11, 12^. Moreover, negative Δ*C*_p_ values are observed for specific protein–DNA interactions^39^, indicating the AmtB–GlnK interaction is highly specific. The sign of Δ*C*_p_ also suggests a change in polar solvation^36, 40^, which is consistent with desolvation of the exposed T-loop of GlnK upon insertion into the ammonia conducting channels of AmtB subunits. Although deciphering the contribution of conformation equilibria and changes in solvation in Δ*C*_p_ warrants further study, the thermodynamic parameters indicate the molecular interaction between AmtB and GlnK is driven by enthalpy and that they adopt flexible conformations.

### Lipids with different headgroups binding to AmtB–GlnK

**Fig. 2:**
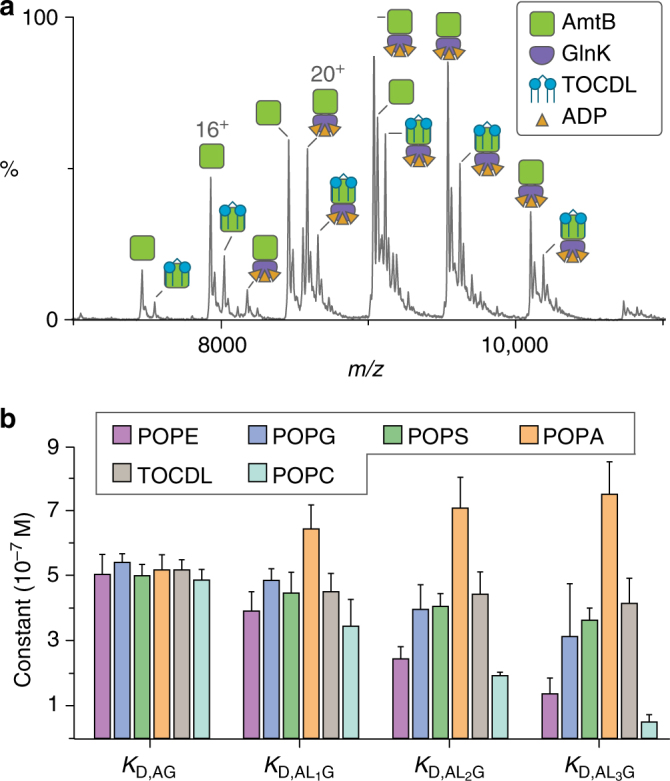
Native mass spectrometry reveals individual lipid-binding events can allosteric modulate the interaction between GlnK and AmtB. **a** Representative mass spectrum of AmtB–GlnK at 2 μM in buffer containing 50 µM ADP and 20 µM 1,1′,2,2′-tetraoleoyl-cardiolipin (TOCDL). **b** Plot of equilibrium dissociation constants (*K*_D_) for GlnK binding to either apo AmtB or AmtB bound to phosphatidylethanolamine (PE), phosphatidylglycerol (PG), phosphatidylserine (PS), and phosphatidic acid (PA) containing 1-palmitoyl-2-oleoyl (PO, 16:0−18:1) tails, and TOCDL at 298 K (see Supplementary Fig. 7 for *K*_D_ abbreviations). Reported are the average and s.e.m. from repeated measurements (*n* = 3)

**Fig. 3:**
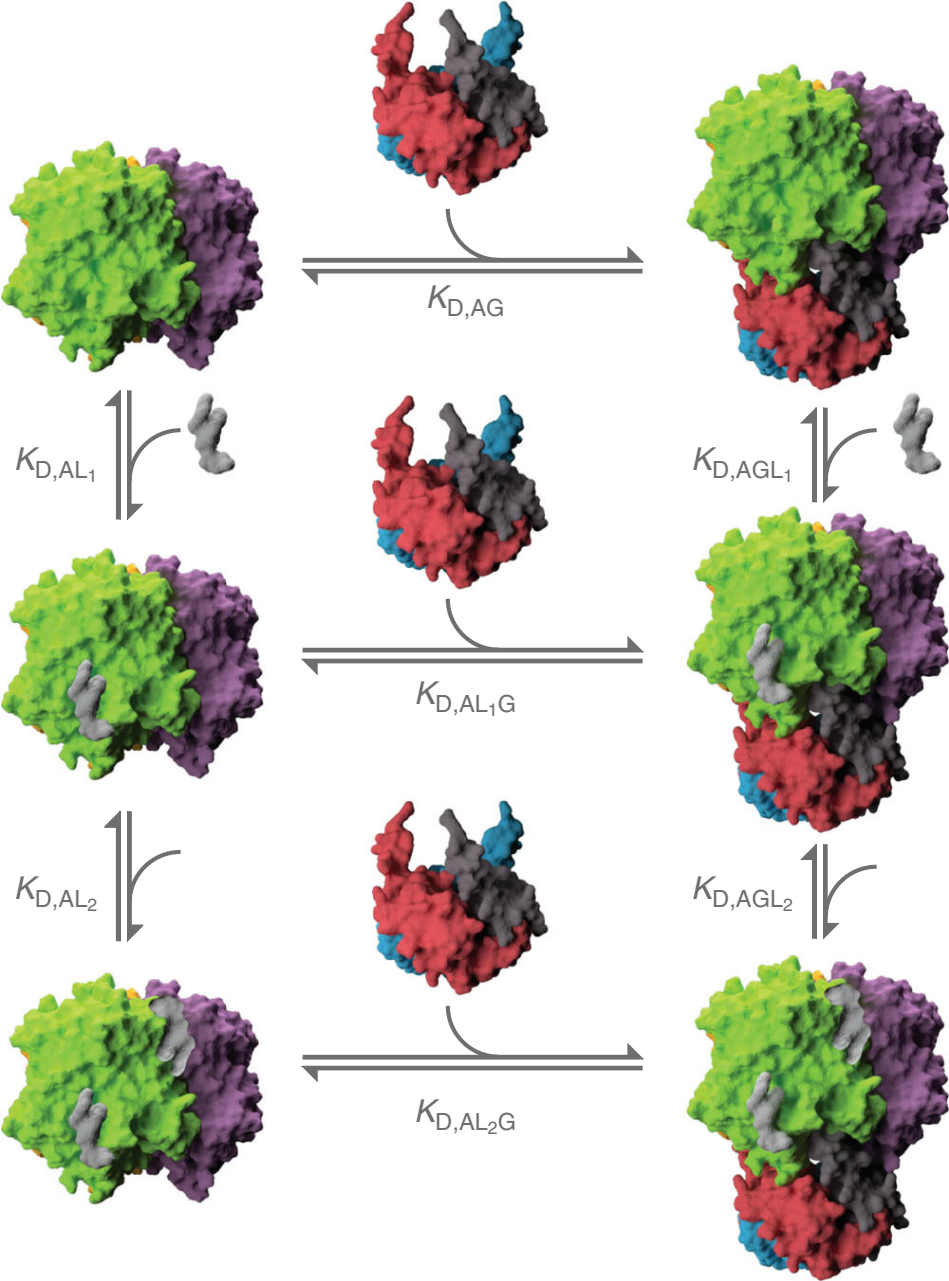
Equilibrium-coupled-binding model for AmtB-binding GlnK and/or lipids. AmtB (green and purple), GlnK (red, blue, and gray), and representative phospholipid (silver) are included in the model. AmtB and GlnK are shown in surface representation (PDB 2NS1). ADP is not shown for clarity

To address whether individual lipid-binding events to AmtB could indirectly influence the interaction with GlnK, we used native MS since it has the ability to resolve individual lipid-binding events unlike other biophysical techniques. Similar to the studies above, the mass spectrometer was tuned to obtain resolved mass spectra while minimizing activation of the complex thereby preserving non-covalent interactions (Supplementary Fig. 5). Mass spectra were recorded under optimized instrument settings for the AmtB–GlnK complex titrated with cardiolipin (TOCDL, 1,1′,2,2′-tetraoleoyl-cardiolipin), phosphatidic acid (PA), phosphatidylethanolamine (PE), phosphatidylglycerol (PG), phosphatidylserine (PS), or phosphatidylcholine (PC) containing 1-palmitoyl-2-oleoyl (PO, 16:0–18:1) tails (Fig. 2 and Supplementary Fig. 6). Although POPC is not native to *E. coli* membranes, it was included since it is a commonly used lipid. Again, an ADP concentration of 50 μM was selected to yield an approximate equal abundance of species enabling simultaneous measurements of lipid-binding events to both AmtB and AmtB–GlnK. The addition of lipids to the AmtB–GlnK complex resulted in lipids binding to both AmtB and AmtB–GlnK (Fig. 2a and Supplementary Fig. 6). Importantly, no lipid-binding events to GlnK were observed in all lipid titrations indicating that the observed lipid-binding events to AmtB and AmtB–GlnK are specific. Equilibrium-binding constants were determined by deconvoluting mass spectra^41^ recorded for AmtB–GlnK titrated with different lipids and fitting the data globally to an equilibrium-coupled AmtB–GlnK lipid-binding model (Fig. 3, Supplementary Fig. 7, and Supplementary Table 2). Importantly, for all lipids, the *K*_D,AG_ values were similar to our previous measurement in the absence of lipid, validating our equilibrium AmtB–GlnK lipid model (Fig. 3).

A range of modulation of the equilibrium dissociation constant (*K*_D,AL*n*G_) for AmtB bound to *n* lipids (AL*n*) binding to GlnK (G) was observed (Fig. 2). POPC, POPE, and POPG exhibited a decrease in *K*_D,AL*n*G_ with each consecutive binding event. Of the three, POPC had displayed the largest decrease in *K*_D,AL*n*G_ followed by POPE. POPC differs from POPE by three methyl groups and these additional moieties likely support binding of this lipid to either a unique site or binding mode, which enhances binding to GlnK. In contrast, an opposite trend was observed for POPA with each binding event weakening the molecular interaction between AmtB and GlnK. On the other hand, TOCDL and POPS had little effect on *K*_D,AL*n*G_. Taken together, these results demonstrate that individual lipid-binding events can indirectly affect the molecular interaction between AmtB and GlnK, presumably by their particular binding mode or position.

### Lipids with different tails binding to AmtB–GlnK

As individual lipid-binding events allosterically modulate the interaction between AmtB and GlnK, we calculated the coupling factors for the different lipids investigated (see “Methods” section). For POPS and TOCDL, the coupling factors exhibited neutral allosteric modulation for all lipid-binding events recorded (Supplementary Table 3). In contrast, POPG and POPE had a consistent increase in the coupling factor with each additional binding event, displaying positive allosteric modulation. More specifically, from a linear fit to the coupling factors as a function of bound lipid yields an increase of 0.4 and 0.3 in the coupling factor per lipid-binding event for POPE and POPG, respectively (Supplementary Fig. 8). Unlike the other lipids investigated, POPA exhibited slight negative allosteric modulation with each lipid-binding event. Remarkably, the lipids investigated, with the exception of TOCDL, differ only by a few atoms in their headgroups yet display a range of allosteric modulation that can be detected by native MS.

**Fig. 4:**
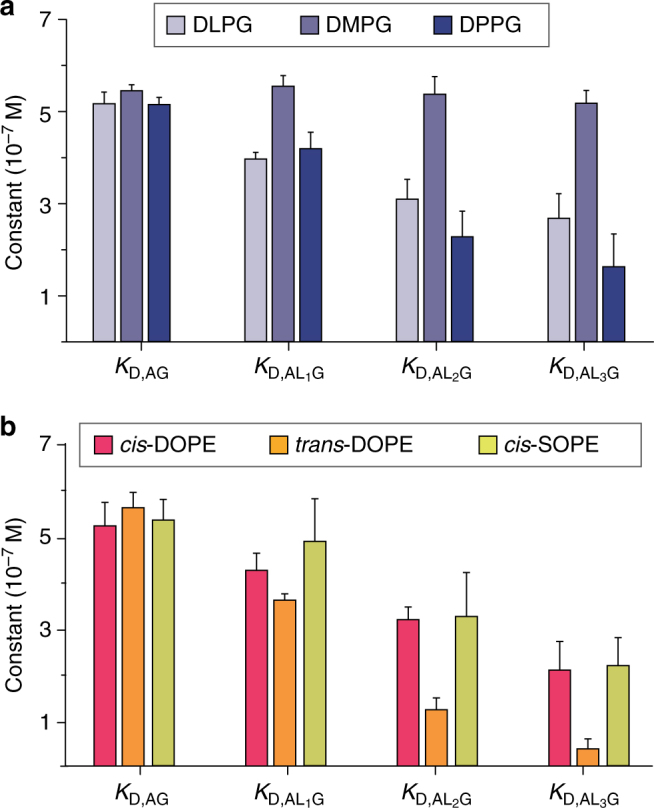
Native mass spectrometry reveals the effect of lipid tail chemistry on allosteric modulation of AmtB–GlnK. **a** Plot of equilibrium dissociation constants (*K*_D_) for GlnK binding apo AmtB and AmtB bound to PG lipids with increasing acyl chain length: 12 (DL, 1,2-dilauroyl), 14 (DM, 1,2-dimyristoyl), and 16 (DP, 1,2-dipalmitoyl). **b** Plot of *K*_D_ for GlnK binding apo AmtB and AmtB bound to PE lipids with different stereochemistry: dioleoyl (DO, 18:1) in *cis* (*cis*-DOPE) or *trans* configuration (*trans*-DOPE), and 1-stearoyl-2-oleoyl (SO, 18:0–18:1) in *cis* configuration (*cis*-SOPE). Reported are the average and s.e.m. from repeated measurements (*n* = 3)

Given the results for lipids with similar tails but different headgroups, we next investigated the effect of varying tail lengths of PG to allosterically modulate the AmtB–GlnK complex. PG was selected as we have previously determined the crystal structure of AmtB in complex with PG and it displayed positive allosteric modulation^14^. In addition, a mutant form of AmtB, where two residues are mutated (N72A/N79A) to abolish a specific PG site observed in the X-ray structure, resulted in reduced gas-phase stabilization of the channel by this lipid^14^ and a different thermodynamic signature observed for binding PG^31^. Equilibrium-binding constants and coupling factors for PG lipids with different tail lengths were determined using the same procedure used for lipids with different headgroups (Fig. 4a and Supplementary Tables 4–5). Consistent with POPG, PG harboring tail lengths of 12 and 16 carbons exhibited positive allosteric modulation. Surprisingly, PG containing 14 carbon tails, in between the lipid tail lengths studied, displayed neutral allosteric modulation. This unexpected result suggests that specific PG-binding site(s) exhibit specific requirements for the lipid tails to elicit an allosteric effect.

Intrigued by the effect of lipid tail length on PG, we then studied the impact of the stereochemistry of monounsaturated carbon bonds within the lipid tails of PE on the AmtB–GlnK complex. In bacteria, the stereochemistry of the monounsaturated double bond is predominantly in the *cis* configuration, which is more abundant at lower growth temperatures, where it increases membrane fluidity^42, 43^. In contrast, the *trans* configuration increases membrane rigidity and decreased permeability to solutes, and its abundance is increased at elevated temperatures^43, 44^. Given the physiological relevance of lipid tail stereochemistry, the first PE lipids we investigated had dioleoyl (DO, 18:1) tails in either the *cis* or *trans* configuration. Equilibrium-binding constants and coupling factors for these lipids were determined in a similar fashion to the other lipids investigated (Fig. 4b and Supplementary Tables 4–5). Interestingly, DOPE in the *trans* configuration, which retains a slight kinked conformation relative to saturated lipids^43^, displayed greater positive allosteric modulation with a coupling factor of 5.28 ± 1.82 for the third bound lipid, representing the largest coupling factor reported in our study (Supplementary Table 5). In contrast, the allosteric modulation for the *cis* configuration, which introduces a kink in the lipid tail, was not as pronounced with a coupling factor of 1.71 ± 0.31 for the third bound lipid. Considering that the *trans* configuration closely resembles that of a saturated carbon–carbon bond, we next examined PE with 1-stearoyl-2-oleoyl (SO, 18:0, 18:1), which contains saturated and *cis*-monounsaturated lipid tails (SOPE). The coupling factor for SOPE did not fall between the two DOPE lipids studied but was similar to *cis*-DOPE. Taken together, these results reveal an exquisite selectivity toward not only the headgroup of lipids, but also the length and stereochemistry of lipid tails.

## Discussion

**Fig. 5:**
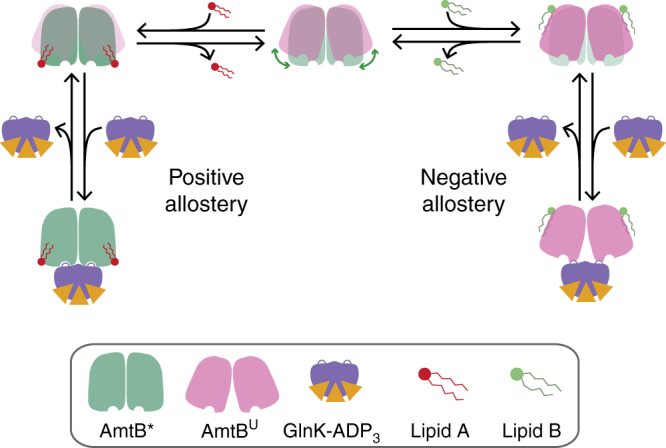
A plausible model for the allosteric modulation of the AmtB–Glnk by individual lipid-binding events. AmtB interconverts between two conformations (middle). Binding of specific lipids may stabilize either conformation. Those that stabilize AmtB*, the state that resembles when bound to GlnK, will lead to positive allosteric modulation. In contrast, lipids that preferentially stabilize AmtB^U^, the state that represents the uncomplexed conformation, will lead to reduced binding affinity to GlnK or negative allostery

Originating in 1965^45^, allostery has emerged as an underlying theme in biological macromolecules (for review see refs. ^46, 47^). Here we demonstrate for the first time that individual lipid-binding events can indeed allosterically modulate integral membrane and soluble protein interactions. Given that we have identified allostery, we propose a plausible mechanism to rationalize the allosteric effects observed (Fig. 5). In the simplest scenario, AmtB is interconverting between two conformations that are observed by X-ray crystallography (Supplementary Fig. 1c): in the uncomplexed state (AmtB^u^) or when complexed with GlnK (AmtB*). In the case of positive allosteric modulation, binding of lipid(s) to AmtB shifts the Boltzmann distribution of the interconverting states of AmtB by preferentially stabilizing (lowering *∆G*) the AmtB* state. As this state resembles that when bound to GlnK, it will exhibit much higher affinity for binding GlnK giving rise to positive allosteric modulation. In contrast, the negative allosteric modulation observed for POPA could be rationalized by preferentially stabilizing the AmtB^u^ state, and since this state resembles that of the uncomplexed state, it will exhibit reduced affinity for GlnK giving rise to negative allostery.

In summary, we have shown that the interaction between AmtB and GlnK is associated with changes in equilibria of conformers and solvation. In addition, we demonstrate that individual lipid-binding events can influence integral membrane protein and soluble protein interactions, using the AmtB–GlnK complex as a model system. Interestingly, lipids with similar tails but different headgroups allosterically modulate the AmtB–GlnK complex in positive, neutral, and negative fashions. The surprising result that variable lipid tail lengths of a PG and the stereochemistry of PE tails can alter the degree of allosteric modulation illustrates that specific lipid-binding site(s) are exquisitely selective for both lipid headgroup and tail. Although a small number of lipid tail lengths and stereochemistry were examined here, by extrapolation it is likely that other modifications of lipid tails will display different degrees of allosteric modulation for protein–protein interactions. Moreover, an underlying question remains regarding the location of the bound lipids and how they induce their allosteric effect. Addressing this question is a monumental challenge as echoed by our limited knowledge in this area. However, in our native MS studies, the lipids may be binding to specific site(s) selective for headgroup and/or lipid tails, or binding to similar non-specific site(s). In closing, this is the first report demonstrating that individual lipid-binding events can have a large dependence on the chemistry of the lipid in modulating membrane protein structure and function, including allosterically modulating protein–protein interactions.

## Methods

### Purification of AmtB–GlnK complex

AmtB wild-type and Strep-tag II-tagged GlnK (stGlnK) was expressed and purified as previously described^14, 31^. In brief, stGlnK was expressed from pET28b (Novagen) in *Escherichia coli* ArcticExpress (DE3) RIL (Agilent Technologies), and protein expression was induced with 0.1 mM 1-thio-β-D-galactopyranoside (IPTG) and grown overnight at 20 °C. Cell pellets of GlnK were resuspended in NHA buffer (50 mM Tris pH 7.4 at room temperature, 300 mM sodium chloride, 20 mM imidazole, and 10% glycerol). The cells were lysed with 4–5 passes through a Microfluidics M-110P microfluidizer at 20,000 psi and then clarified by centrifugation (30 min at 30,000 × *g* at 4 °C). The filtered supernatant containing recombinant Strep-tag II-tagged GlnK was then applied onto a StrepTrap HP 5 mL column (GE Healthcare) and eluted with the same buffer containing 2.5 mM D-desthiobiotin. The peak fractions containing Strep-tag II-tagged GlnK were pooled, concentrated, and loaded onto a HiLoad 16/600 Superdex 75 pg column (GE Healthcare) equilibrated in GF buffer (20 mM Tris pH 7.4 at room temperature, 100 mM sodium chloride, and 10% glycerol). AmtB was expressed as TEV protease cleavable N-terminal fusion to maltose-binding protein (MBP) preceded by a secretion signal peptide (pelB) and 10x His-tag from pET15b (Novagen) in *Escherichia coli* C41(DE3) (Lucigen). Expression of AmtB was induced with 0.5 mM IPTG and cells were grown overnight at 20 °C. Cell pellets of AmtB were resuspended and lyszed as described for stGlnK. Membranes were pelleted by centrifugation (2 h at 100,000 × *g* at 4 °C) and AmtB was extracted overnight with 5% octyl glucoside. The supernatant was filtered before loading onto a 5 mL HisTrap-HP column (GE Healthcare) equilibrated in buffer NHA-DDM (200 mM sodium chloride, 10% glycerol, 20 mM imidazole, 0.025% DDM and 50 mM Tris pH 7.4 at room temperature). After the clarified supernatant was loaded, the column was initially washed with 40–50 mL of NHA-DDM supplemented with 1% OG followed by several column volumes of NHA-DDM until a steady baseline was reached. AmtB was eluted with a linear gradient to 100% in two column volumes of NHB-DDM (100 mM sodium chloride, 10% glycerol, 500 mM imidazole, 0.025% DDM and 50 mM Tris pH 7.4 at room temperature). Peak fractions were pooled and injected onto a HiPrep 26/10 desalting column (GE Healthcare) equilibrated in NHA-DDM. Peak fractions were pooled and supplemented with 5 mM 2-mercaptoethanol (BME) and His-tagged TEV and incubated overnight at 4 °C. After overnight incubation, the sample was filtered and passed back over a 5 mL HisTrap HP column equilibrated in NHA-DDM. Flow-through containing the untagged AmtB was collected and concentrated using a 100 kDa MWCO concentrator. The Y51F point mutation was introduced into GlnK as this residue can be modified with uridine by uridylyl transferase GlnD and prevent the interaction with AmtB^8, 12, 48^. Unless otherwise stated, all purification steps were carried out at 4 °C. To generate the AmtB–GlnK complex, purified tagless AmtB and stGlnK were mixed at a molar ratio of 1:3 in GF buffer (50 mM Tris pH 7.4 at room temperature, 100 mM sodium chloride, 10% glycerol, 0.025% DDM, and 1 mM adenosine diphosphate (ADP)) and incubated overnight. The mixture of AmtB and stGlnK was loaded onto a StrepTrap HP 5 mL column (GE Healthcare) to capture both AmtB in complex with stGlnK and stGlnK, and flow through contained non-functional AmtB. Bound protein was eluted with the same buffer containing 2.5 mM D-desthiobiotin, concentrated, and loaded onto a Superdex 200 Increase 10/300 GL column (GE Healthcare) equilibrated in GF buffer supplemented with 0.5% C_8_E_4_ instead of DDM. Peak fractions containing the AmtB–stGlnK were pooled, concentrated, flash frozen in liquid nitrogen, and stored at −80 °C.

### Protein quantification

Soluble and membrane protein concentration in the buffer not containing ADP was determined with the DC Protein Assay kit (Bio-Rad) using bovine serum albumin as the standard. For quantification of samples containing ADP, which interferes with protein assays, aliquots of the purified AmtB–GlnK complex were thawed at room temperature, immediately mixed with 0.25 volumes of 5× Laemmli sample buffer^49^, and incubated at room temperature for 30 min. Heating was avoided as it promotes aggregation of AmtB. The prepared samples were loaded on a precast 12% acrylamide gel (TGX, 10 well, BioRad). Included on each gel was one well with pre-stained size markers (BioRad), and at least two wells loaded with a standard mixture of purified AmtB and stGlnK at a molar ratio of 1:1. Gels were run at 60 V during stacking and at 100 V once sample was in the resolving gel at room temperature. After separation, each gel was rinsed in water for 15 min, stained with 0.025% Coomassie brilliant blue R-250 (Sigma B-0149) in 10% acetic acid using a rapid, hot-staining protocol^50^ followed destaining overnight in a 100 mL portion of 10% acetic acid. General procedures for quantitative densitometry followed the recommendations of Gassmann and colleagues^51^. The gel was placed on a Ricoh 4504 scanner, imaged in transmittance mode at a resolution of 400 dpi, and stored as a 16-bit greyscale TIFF image. For densitometry analysis, each file was opened in ImageJ^52^ and the original greyscale values were converted to optical density (OD) values. Individual full-width lanes were then defined, their OD profiles calculated, and the integrated stain density summed for the AmtB and GlnK bands. The amount of AmtB or GlnK protein in each lane was calculated from the stain density in the lane, with reference to the average stain density of the AmtB or GlnK band in lanes loaded with the known standard mix of AmtB and GlnK. The protein concentration for AmtB and GlnK was the average of the values determined from at least two separately Coomassie-stained gels.

### Surface plasmon resonance

SPR experiments were performed using a Biacore 3000 optical biosensor (GE Healthcare/Biacore AB) with CM3 sensor chip. Immobilization of GlnK was conducted at 25 ˚C using amine coupling kit (GE Healthcare) at a flow rate of 10 µL min^−1^ in PBS (8.06 mM Na_2_HPO_4_ and 1.94 mM KH_2_PO_4_, 2.7 mM KCl, 137 mM NaCl, pH 7.4). In order to generate low-density GlnK sensor, the chip surface was pretreated by sequential injection of 40 µL of activation solution (1:1 mixture of 0.4 M EDC and 0.1 M NHS) and 40 µL of deactivation solution (1.0 M ethanolamine, pH 8.5). The pretreated surface was activated again for 2 min followed by 1 min injection of stGlnK in buffer (2.5–5 µg ml^−1^ in 10 mM sodium acetate, pH 5.5), and then washed with 200 mM ammonium acetate (pH 7.4) until no further decline in signal response. Using this procedure, GlnK surface with different densities ranging from 150 to 400 RU was produced. A flow cell with the same treatment but without coupled protein was used as a reference surface. The sensor chip was further stabilized with 10 injections of binding buffer (200 mM ammonium acetate, pH 7.4, 50 µM ADP, 0.5% C_8_E_4_) followed by 1 h of continuous flow at 30 µL min^−1^. Binding experiments were performed at a flow rate of 30 µL min^−1^ at different temperatures (13, 17, 21, 25, and 29 ˚C) using freshly buffer-exchanged AmtB in binding buffer at a concentration range of 0.05–1.4 µM. Reference and buffer corrected SPR responses were collected, and the data were analyzed using Biacore evaluation software. The kinetic parameters and *K*_D_ value were determined for each temperature by fitting the sensorgram series to a Langmuir 1:1 binding model.

### Preparation and titration of ADP or phospholipids

A stock of ADP was prepared by dissolving the ammonium salt form of ADP in water followed by adjusting the pH with ammonium hydroxide to 7.0. Serial dilutions into MS buffer (200 mM ammonium acetate, 0.5% C8E4, and pH adjusted to 7.4 with ammonium hydroxide) supplemented with 5 mM BME was performed to prepare ADP at certain concentrations. Phospholipids were prepared as previously described^31^. In brief, stock solutions of each phospholipid were resuspended in chloroform, aliqouted, and chloroform was removed by a steady stream on nitrogen gas. Lipid films were placed under vacuum overnight to prepare to resuspend in MS buffer supplemented with 5 mM 2-mercaptoethanol. Phospholipid concentration was determined by phosphorus analysis.

### Preparation of AmtB–GlnK complex for native MS

A frozen aliquot of purified AmtB–GlnK complex was thawed on ice prior to buffer exchange into MS buffer supplemented with a desired concentration of ADP following established methods^53^.

### Native MS

Native MS was performed on a Synapt G1 HDMS instrument (Waters corporation) with a 32 k RF generator. AmtB–GlnK samples were at a final concentration of 2 µM. Instrument parameters were tuned to maximize ion intensity and simultaneously preserve the native-like state of AmtB. The instrument was set to a capillary voltage of 1.7 kV, sampling cone voltage of 100 V, extractor cone voltage of 10 V, and argon flow rate at 7 mL min^−1^ (5.2 × 10^–2^ mbar). The T-wave settings for trap (300 ms^−1^/2.0 V), IMS (300 ms^−1^/20 V), and transfer (100 ms^−1^/10 V), source temperature (110 °C) trap collision voltage (20 V) and transfer collision voltage (160 V) and trap bias (35 V) were also optimized.

### Native MS data analysis

Native MS data were processed using the software program Pulsar^54^ and deconvoluted using Unidec^55^ software with the following settings: no smoothing, *m/z* range 6000–12,000, charge range 10–30, mass sampling of 5 Da, and peak FWHM of 1. The intensities of apo AmtB (*A*) and AmtB–GlnK (AG) and AmtB (AL_*n*_) and AmtB–GlnK (AGL_*n*_) bound to L_*n*_ lipid(s) were converted to mole fraction for a given lipid titration. Notably, the non-specific ADP adducted peaks of AmtB and AmtB–GlnK and those bound to lipid(s) were taken into account when converting to mole fraction. The interaction between AL_*n*_ and GlnK (*G*) is dependent on the apparent equilibrium association constant (*K*_A,AL*n*G_):

1 $${K}_{{\mathrm{A}},{\mathrm{AL}}n{\mathrm{G}}} = \frac{{[{\mathrm{AL}}_nG]}}{{[{\mathrm{AL}}_n][G]}}$$

Where *n* is an integer starting at 0 in the case of apo. And for a lipid binding to A:

2 $${K}_{{\mathrm{A}},{\mathrm{AL}}n} = \frac{{[{\mathrm{AL}}_n]}}{{\left[ {{\mathrm{AL}}_{n - 1}} \right]\left[ {L} \right]}}$$

And binding event to AG:

3 $${K}_{{\mathrm{A}},{\mathrm{AGL}}n} = \frac{{\left[ {\mathrm{AGL}_n} \right]}}{{\left[ {\mathrm{AGL}_{n - 1}} \right]\left[ L \right]}}$$

The total AmtB (*A*_total_) in the system can be represented as:

4 $$\left[ {{A}_{{\mathrm{total}}}} \right] = \left[ {A} \right] + \mathop {\sum }\limits_{i = 1}^n \left[ {{\mathrm{AL}}_i} \right] + \left[ {{\mathrm{AG}}} \right] + \mathop {\sum }\limits_{i = 1}^n \left[ {{\mathrm{AGL}}_{i}} \right]$$

substituting Eqs. (1–3) into (4):

5 $$\left[ {{A}_{{\mathrm{total}}}} \right] = \left[ {A} \right] + \mathop {\sum }\limits_{i = 1}^n \left[ {A} \right]\left[ {L} \right]^i\mathop {\prod }\limits_{j = 1}^i {K}_{{\mathrm{A}},{\mathrm{AL}}j} + {K}_{{\mathrm{A}},{\mathrm{AL}}_0{\mathrm{G}}}\left[ {A} \right]\left[ {G} \right] \\ + \mathop {\sum }\limits_{i = 1}^n {K}_{{\mathrm{A}},{\mathrm{AL}}i{G}}\left[ {A} \right]\left[ {\mathrm{G}} \right]\left[ {L} \right]^i\mathop {\prod }\limits_{j = 1}^i {K}_{{\mathrm{A}},{\mathrm{AL}}j}$$

Equation (5) can be rearranged to calculate the mole fraction of AL*n* (*F*_AL*n*_):

6 $${F}_{{\mathrm{AL}}n} = \frac{{\left[ {L} \right]^n\mathop {\prod }\nolimits_{j = 1}^n {K}_{{\mathrm{A}},{\mathrm{AL}}j}}}{{\left[ {{A}_{{\mathrm{total}}}} \right]}}$$

And for AGL*n* (F_AGL*n*_):

7 $${F}_{{\mathrm{AGL}}n} = \frac{{{K}_{{\mathrm{A}},{\mathrm{AL}}n{\mathrm{G}}}\left[ {G} \right]\left[ {L} \right]^n\mathop {\prod }\nolimits_{j = 1}^n {K}_{{\mathrm{A}},{\mathrm{AL}}j}}}{{\left[ {{A}_{{\mathrm{total}}}} \right]}}$$

The free concentration of *G* was calculated as follows:

8 $$\left[ {G} \right] = \left[ {{G}_{{\mathrm{total}}}} \right] - \left\{ {\left[ {{A}_{{\mathrm{total}}}} \right] \cdot \left( {{F}_{{\mathrm{AG}}} + \mathop {\sum }\limits_{i = 1}^n {F}_{{\mathrm{AGL}}i}} \right)} \right\}$$

And for the free concentration of *L*:

9 $$\left[ {L} \right] = \left[ {{L}_{{\mathrm{total}}}} \right] - \left\{ {\left[ {{A}_{{\mathrm{total}}}} \right] \cdot \left( {\mathop {\sum }\limits_{i = 1}^n i{F}_{{\mathrm{AL}}i} + \mathop {\sum }\limits_{i = 1}^n i{F}_{{\mathrm{AGL}}i}} \right)} \right\}$$

The above binding model (Fig. 3) was coded into Python (http://www.python.org) and made use of libraries scipy^56^, numpy^57^, and matplotlib^58^. The binding model was globally fit to the mole fraction data collected at a given temperature through minimization of the pseudo-χ^2^ function:

10 $$\chi ^2 = \mathop {\sum }\limits_{i = 0}^n \mathop {\sum }\limits_{j = 0}^d \left( {{F}_{{\mathrm{AL}}i_j}^{{\mathrm{exp}}} - {F}_{{\mathrm{AL}}i_j}^{{\mathrm{calc}}}} \right)^2 + \left( {{F}_{{\mathrm{AGL}}i_j}^{{\mathrm{exp}}} - {F}_{{\mathrm{AGL}}i_j}^{{\mathrm{calc}}}} \right)^2$$

where *n* is the total number of lipids bound and *d* is the total number of data points.

In the above binding model, all equilibrium association constants are treated as a separate variable in the fitting routine. The coupling factor (α_*i*_) for a given number of lipids bound (*i*) was calculated as follows:

11 $$\propto _i = \frac{{{K}_{{\mathrm{A}},{\mathrm{AGL}}i}}}{{{K}_{{\mathrm{A}},{\mathrm{AGL}}(i - 1)}}}$$

Thermodynamic parameters for the AmtB–GlnK complex in the absence of lipid were determined using the nonlinear form of the “van’t” Hoff equation:^59^

12 $$\ln \left( {{{K}}_{\mathrm{A}}} \right) = \frac{{\Delta {{H}}_{{T}_{\mathrm{o}}} - {T}_{\mathrm{o}}\Delta {C}_{\mathrm{p}}}}{R}\left( {\frac{1}{{{T}_{\mathrm{o}}}} - \frac{1}{{\mathrm{T}}}} \right) + \frac{{\Delta {C}_{\mathrm{p}}}}{R}\ln \left( {\frac{{T}}{{{T}_{\mathrm{o}}}}} \right) + \ln \left( {{K}_{\mathrm{o}}} \right)$$

where *K*_A_ is the equilibrium association constant, *K*_o_ is the equilibrium association constant at the reference temperature (*T*_o_), Δ*H* is the standard enthalpy at *T*_o_, Δ*C*_p_ is the change in heat capacity at constant pressure, and *R* is the universal gas constant. The average and standard error of the mean was determined by fitting the above equation coded into Python to experimental data for each replicate with *T*_o_ equal to 298 K.

### Data availability

The data that support the findings of this study are available within the article and Supplementary Information. Other data and binding model Python code are available from the corresponding author on reasonable request.

## Electronic supplementary material

Supplementary Information
