# Supplementary material for: Allosteric modulation of protein-protein interactions by individual lipid binding events
Source: Nat Commun. 2017 Dec 19;8:2203. doi: 10.1038/s41467-017-02397-0 (PMC5736629; doi:10.1038/s41467-017-02397-0)
Supplement: Supplementary file 1 — Supplementary Information [file 41467_2017_2397_MOESM1_ESM.pdf]

Supplementary Figures

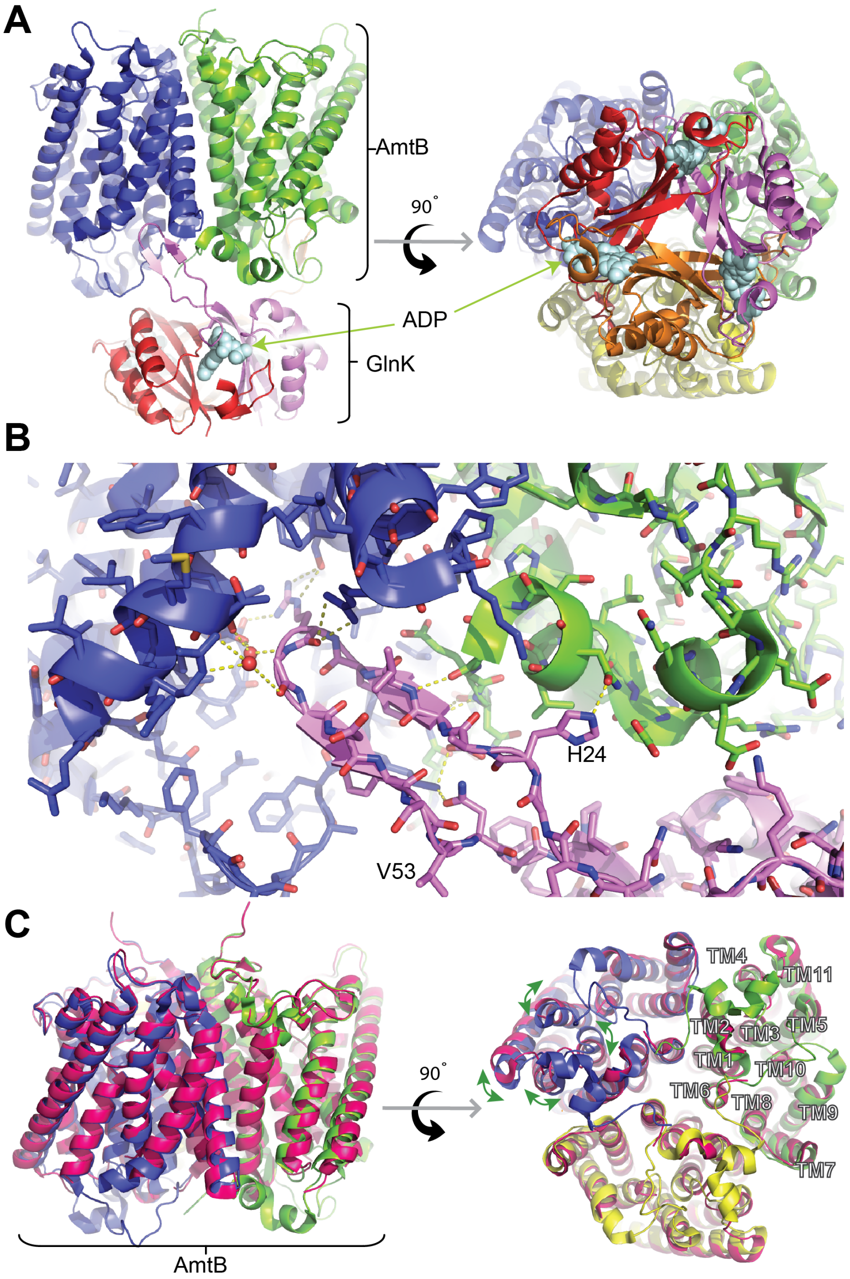

Supplementary Figure 1. Structural overview of AmtB and in complex with GlnK. A) Shown is the crystal structure of AmtB-GlnK (PDB 2NS1) in cartoon representation and bound ADP as blue spheres. B) Molecular details of the T-loop of GlnK (residues H24-V53) binding AmtB. A total of 13 hydrogen bonds (yellow dashed lines) between AmtB and GlnK per subunit are formed. C) Structural comparison of AmtB-GlnK (PDB 2NS1) and apo AmtB (PDB 4NH2, solid magenta). The eleven transmembrane helices (TM) are labeled. The conformational change (or translation of TM 5, 7, 10, and 11) between the apo and GlnK bound structures is highlighted (green arrows). Images of structures were generated using Pymol.[1]

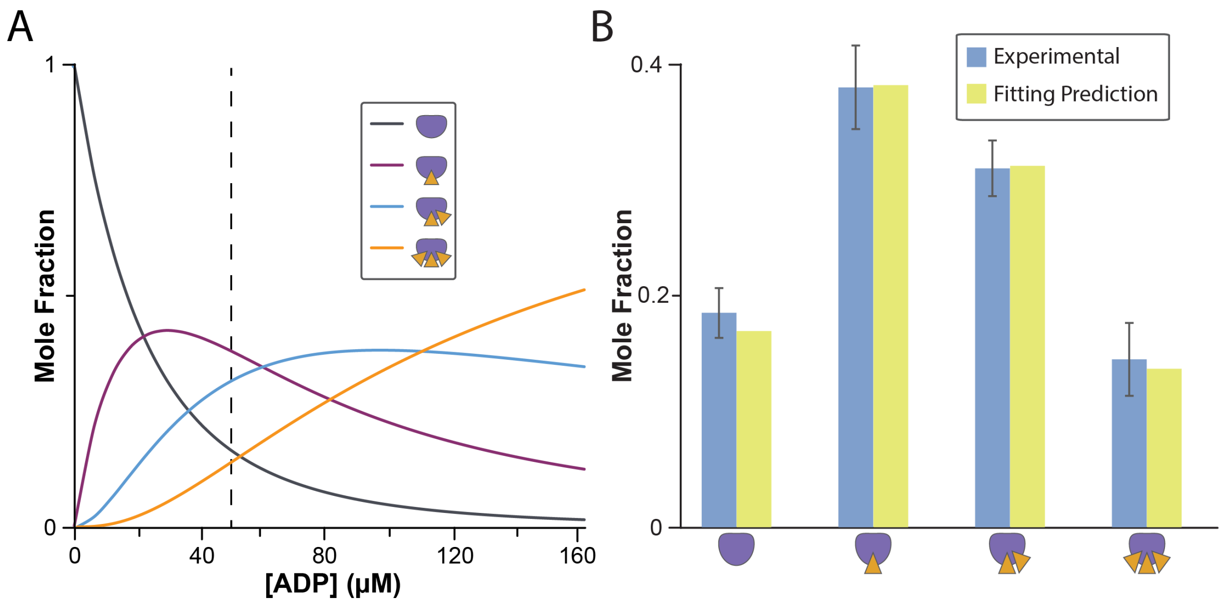

Supplementary Figure 2. Comparison of calculated and experimental mole fraction for GlnK-ADP$_{0\text{-}3}$. A) Plot of the mole fraction of GlnK(ADP)$_{0\text{-}3}$ calculated from the K_D at 298K for binding the first, second, and third ADP molecule at 20 µM, 56 µM, and 100 µM, respectively.[2] The GlnK concentration in the calculation was at 2 µM, which is the same concentration used in our AmtB-GlnK studies. B) Experimental and calculated mole fraction of GlnK·ADP$_{0\text{-}3}$ at 298K and ADP at 46 µM, which is the concentration after subtracting the ADP trapped in AmtB-GlnK-ADP$_3$. Labels are shown as in Figure 2A. Reported experimental values are average and standard error of the mean (s.e.m, *n* = 3)

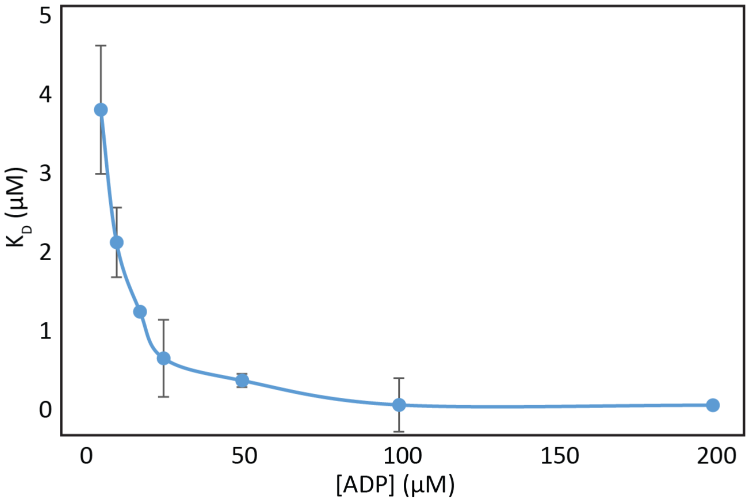

Supplementary Figure 3. Effect of ADP concentration on the equilibrium dissociation constant for the AmtB-GlnK complex. The y-axis represents the total ADP concentration. Reported are average and s.e.m. (n = 2).

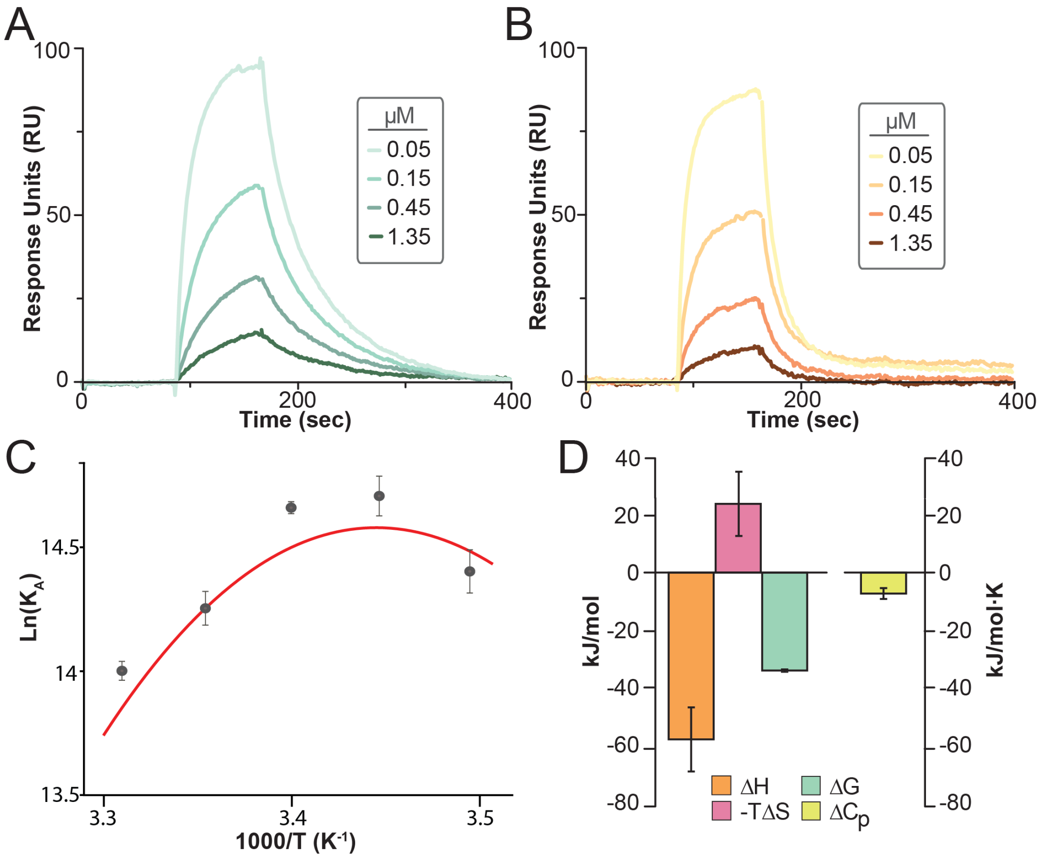

Supplementary Figure 4. Biophysical characterization of the AmtB-GlnK complex by SPR. A-B) Representative sensorgrams of AmtB injected at different concentrations in MS buffer containing 50 μM ADP and 2x CMC C_8E_4 over a sensor surface immobilized with GlnK at 290K (green lines) and at 302K (orange lines). C) van 't Hoff plot for AmtB-GlnK binding determined by SPR at different temperatures (grey dots), and resulting fit of the nonlinear van 't Hoff equation (red line). Reported are the average and s.e.m. (n = 3). D) Thermodynamic parameters for AmtB-GlnK binding derived from van 't Hoff analysis of SPR data. The change in free energy (ΔG) was calculated directly from repeated measurements of $K_{D,AG}$ at 298K, and entropy (ΔS) was back calculated using both ΔH and ΔG (Supplementary Table 1). Reported are average and s.e.m. (n = 3).

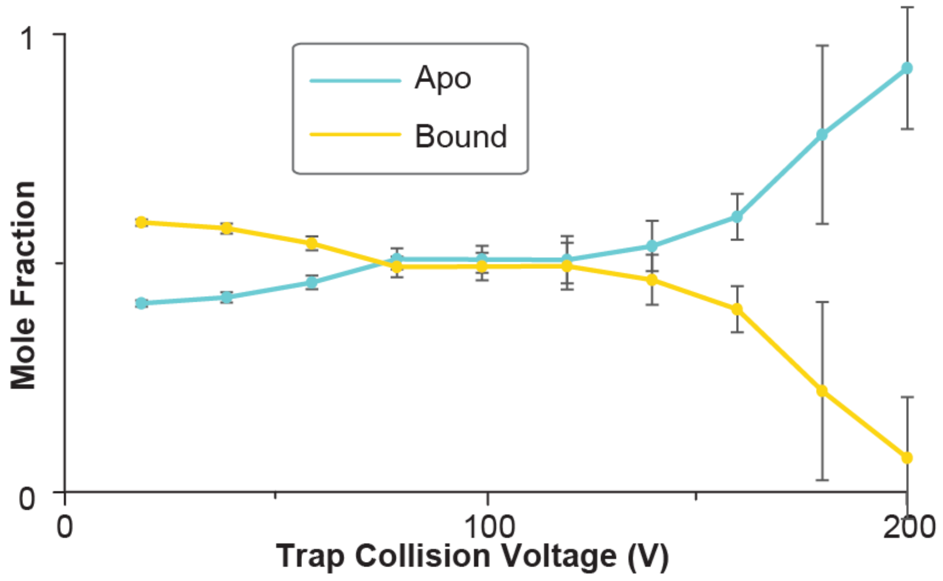

Supplementary Figure 5. Impact of instrument settings on the abundance of apo and lipid-bound AmtB and AmtB-GlnK. Blue lines represent apo mole fractions, whereas yellow lines represent the sum of mole fractions bound to POPE. Instrument setting used in this study corresponds to the first pair of data on the left. Reported are average and s.e.m. (*n* = 3)

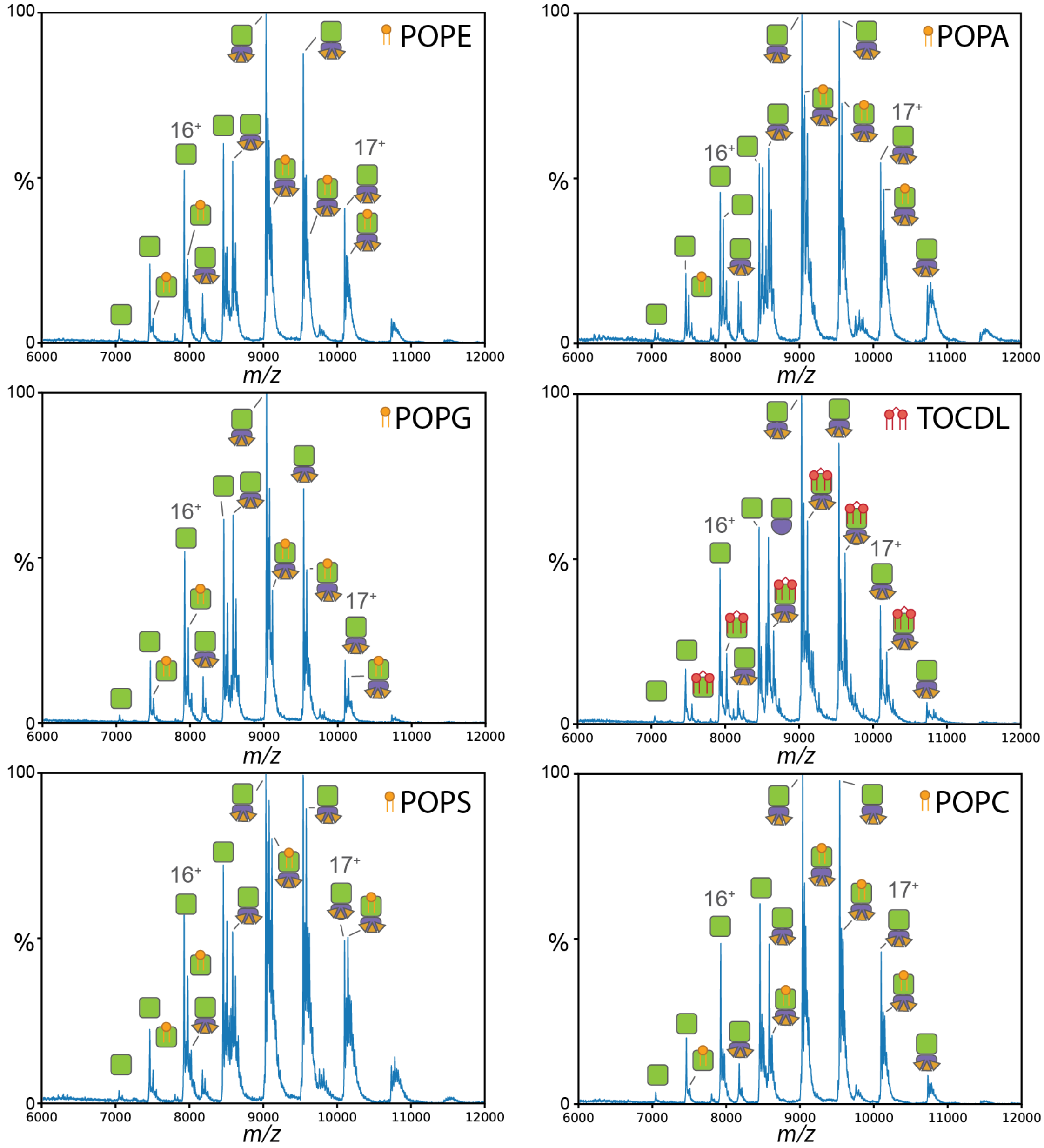

Supplementary Figure 6. Representative mass spectra for AmtB-GlnK titrated with different lipids. Data was collected at 25 °C under instrument setting optimized to preserve non-covalent interactions. Similar mass spectra were observed for all lipids in terms of resolution, charge states and ion abundances. Labels are shown as in Figure 2A.

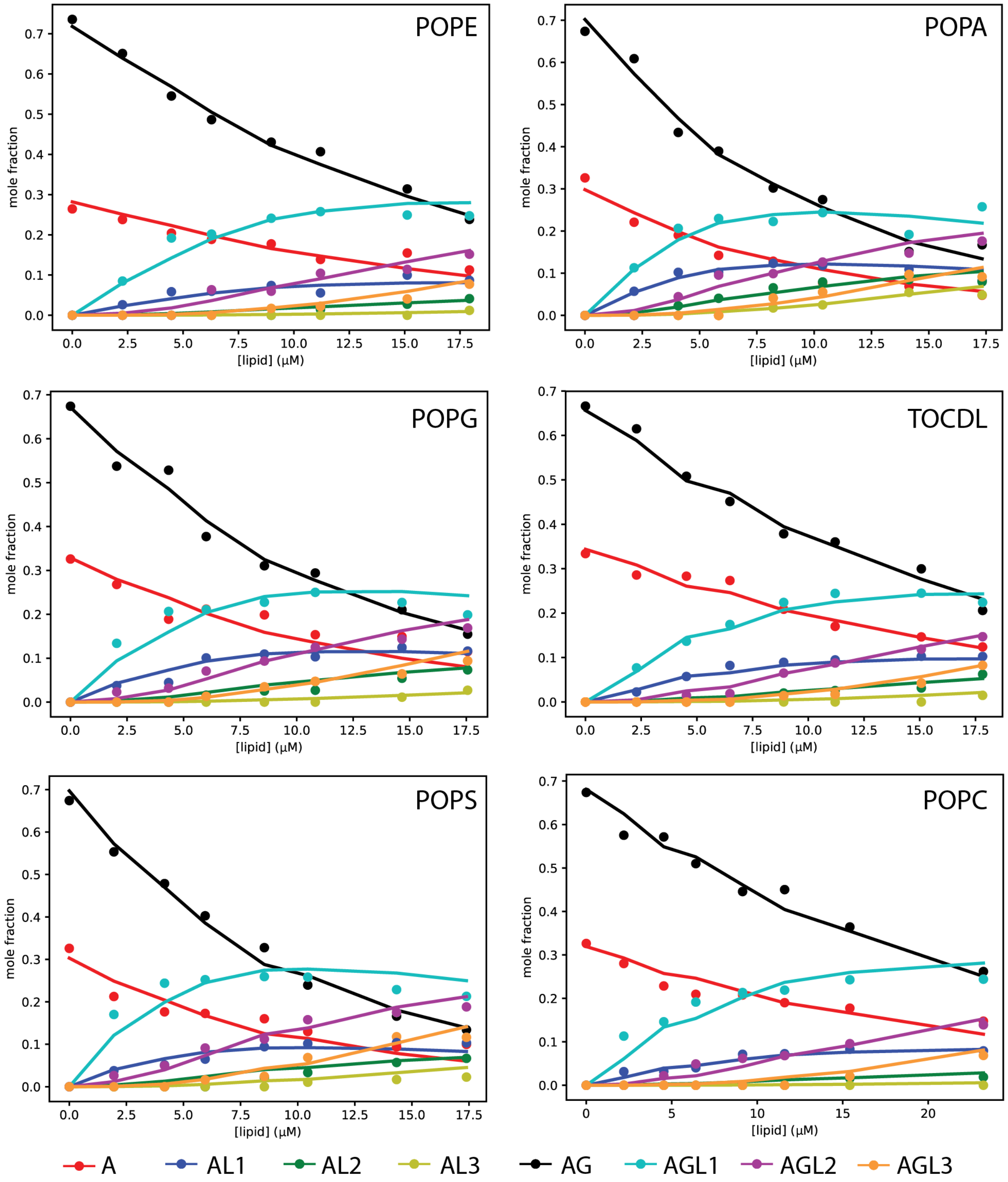

Supplementary Figure 7. Representative plots of mole fraction for apo and lipid bound species of AmtB and AmtB-GlnK as a function of free lipid concentration collected at 25 °C. Experimental data (dots) and resulting fit (solid lines) from AmtB-GlnK-lipid binding model (Supplementary Fig. 7, also see Methods). The legend, located at the bottom, for the different species plotted correspond to A is AmtB, L*n* is phospholipid bound to *n*, G is GlnK, and AL*n* or AGL*n* is AmtB or AmtB-GlnK bound to *n* lipids.

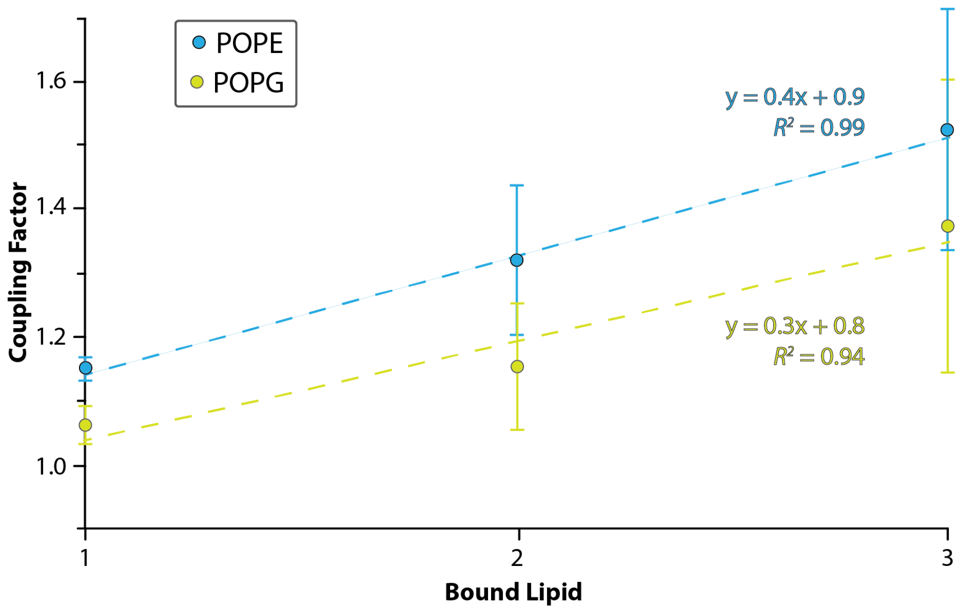

Supplementary Figure 8. Plot of coupling factor as a function of bound lipid for POPE and POPG. The fit of linear line (dashed line) to data and the corresponding equation and R^2 is shown. Reported are average and s.e.m.

Supplementary Tables

Supplementary Table 1. AmtB-GlnK binding constants and thermodynamic parameters determined by nonlinear van 't Hoff analysis and the reference temperature of 298K (n = 4 and 3 for MS and SPR, respectively)

Method	$K_{D,AG}$ (μM)	k_{on} ($\times10^4$ $M^{-1}s^{-1}$)	k_{off} ($\times10^{-2}$ s^{-1})	ΔH (kJ/mol)	ΔC_P (kJ·mol^{-1}·K^{-1})	ΔG (kJ·mol^{-1})	-TΔS (kJ·mol^{-1})	R^2
**MS**	1.12 ± 0.41	-	-	-47.50 ± 8.08	-10.24 ± 4.93	-34.45 ± 0.92	13.05 ± 8.13	0.98
**SPR**	0.65 ± 0.05	5.49 ± 0.44	3.14 ± 0.18	-60.10 ± 11.57	-7.60 ± 1.95	-35.43 ± 0.21	24.78 ± 11.58	0.87

ΔG was calculated directly from K_D, and -TΔS back calculated using ΔG and ΔH. Reported are average and s.e.m.

Supplementary Table 2. Equilibrium dissociation constants and fitting statistics for AmtB-GlnK titrated with different lipids

	POPA	POPC	POPE	POPG	POPS	TOCDL
$K_{D,AL1}$ (μM)	8.11 ± 0.41	29.23 ± 5.99	21.14 ± 0.27	13.48 ± 0.73	10.49 ± 0.97	19.87 ± 1.65
$K_{D,AL2}$ (μM)	18.14 ± 1.18	68.30 ± 4.81	55.28 ± 9.22	33.27 ± 6.39	21.80 ± 1.97	30.18 ± 2.05
$K_{D,AL3}$ (μM)	24.75 ± 2.44	104.29 ± 7.56	96.24 ± 26.99	56.66 ± 11.82	30.43 ± 4.97	33.05 ± 6.01
$K_{D,AG}$ ($\times10^{-8}$M)	51.74 ± 4.63	48.63 ± 3.25	50.38 ± 6.16	54.04 ± 2.67	49.95 ± 3.47	51.74 ± 3.12
$K_{D,ALG}$ ($\times10^{-8}$ M)	64.39 ± 7.35	34.46 ± 8.22	39.12 ± 5.93	48.52 ± 3.56	44.63 ± 6.44	45.05 ± 5.61
$K_{D,AL2G}$ ($\times10^{-8}$ M)	70.75 ± 9.45	19.24 ± 1.12	24.46 ± 3.72	39.65 ± 7.54	40.54 ± 3.95	44.27 ± 6.89
$K_{D,AL3G}$ ($\times10^{-8}$ M)	75.03 ± 9.92	50.31 ± 22.52	13.72 ± 4.82	31.29 ± 16.21	36.27 ± 3.72	41.43 ± 7.76
$K_{D,AGL1}$ (μM)	10.02 ± 0.31	18.86 ± 1.36	16.28 ± 0.40	12.04 ± 0.06	9.18 ± 0.60	16.99 ± 0.17
$K_{D,AGL2}$ (μM)	19.78 ± 0.58	40.32 ± 3.92	33.64 ± 1.24	25.42 ± 1.11	19.85 ± 0.36	29.34 ± 1.42
$K_{D,AGL3}$ (μM)	26.22 ± 2.19	59.52 ± 17.86	47.67 ± 7.02	34.32 ± 3.40	26.68 ± 1.82	29.46 ± 1.68
R^2	0.98	0.99	0.98	0.98	0.98	0.99
χ^2 ($x10^{-5}$)	1.53	2.67	5.96	3.53	3.32	3.38

Reported are the average and s.em. (n = 3)

Supplementary Table 3. Coupling factors (α_n) for AmtB binding to either *nth* lipid and GlnK. Reported are average and s.e.m (n = 3)

Lipid	α_1	α_2	α_3
**POPE**	1.30 ± 0.04	1.63 ± 0.24	2.04 ± 0.37
**POPG**	1.12 ± 0.06	1.30 ± 0.21	1.74 ± 0.46
**POPS**	1.15 ± 0.09	1.09 ± 0.08	1.13 ± 0.10
**POPA**	0.81 ± 0.02	0.92 ± 0.04	0.94 ± 0.04
**TOCDL**	1.17 ± 0.09	1.03 ± 0.06	1.11 ± 0.15
**POPC**	1.52 ± 0.23	1.76 ± 0.31	2.50 ± 0.80

Supplementary Table 4. Equilibrium dissociation constants and fitting statistics for AmtB-GlnK titrated with phosphatidylglycerol with different lipid chain lengths and phosphatidylethanolamine with different lipid stereochemistry

	DLPG	DMPG	DPPG	SOPE	DOPE (cis)	DOPE (trans)
$K_{D,AL1}$ (μM)	22.47 ± 3.23	6.78 ± 0.19	18.53 ± 1.85	16.36 ± 1.39	15.11 ± 0.63	29.69 ± 2.18
$K_{D,AL2}$ (μM)	39.07 ± 9.25	14.64 ± 1.20	63.83 ± 13.20	46.58 ± 9.46	36.92 ± 2.85	159.17 ± 57.32
$K_{D,AL3}$ (μM)	34.03 ± 0.15	20.27 ± 0.12	99.77 ± 45.26	73.61 ± 21.98	57.61 ± 13.27	--
$K_{D,AG}$ (x10^{-8} M)	52.23 ± 2.58	55.07 ± 1.34	52.07 ± 1.58	52.78 ± 4.48	51.67 ± 5.04	55.56 ± 3.27
$K_{D,ALG}$ (x10^{-8} M)	40.07 ± 1.48	56.07 ± 2.35	42.37 ± 3.61	48.19 ± 9.19	42.06 ± 3.75	35.64 ± 1.41
$K_{D,AL2G}$ (x10^{-8} M)	31.30 ± 4.35	54.33 ± 3.87	23.03 ± 5.59	32.08 ± 9.44	31.50 ± 2.69	12.11 ± 2.56
$K_{D,AL3G}$ (x10^{-8} M)	27.07 ± 5.39	52.33 ± 2.84	16.44 ± 7.21	21.50 ± 6.07	20.61 ± 6.21	37.14 ± 2.17
$K_{D,AGL1}$ (μM)	17.13 ± 1.96	6.92 ± 0.33	14.83 ± 0.64	14.55 ± 1.28	12.31 ± 0.39	19.03 ± 0.42
$K_{D,AGL2}$ (μM)	28.60 ± 3.98	14.07 ± 0.35	31.67 ± 1.89	2.85 ± 2.32	27.55 ± 1.09	46.0 0 ± 4.91
$K_{D,AGL3}$ (μM)	29.10 ± 2.27	19.57 ± 0.33	53.43 ± 16.58	45.22 ± 7.56	32.89 ± 2.11	--
R^2	0.98	0.97	0.98	0.96	0.97	0.98
χ^2 (x10^{-5})	6.02	4.18	6.38	13.52	23.98	7.96

Reported are the average and s.e.m. (*n* = 3). -- Indicates species that were not observed leading to erroneously high values.

Supplementary Table 5. Coupling factors (α_n) for AmtB-GlnK complex binding to *n* phosphatidylglycerol lipids with different lipid chain lengths phosphatidylethanolamine with different lipid stereochemistry

Lipid	Chain Length	α_1	α_2	α_3
**DLPG**	12:0	1.30 ± 0.04	1.35 ± 0.26	1.19 ± 0.15
**DMPG**	14:0	0.99 ± 0.05	1.04 ± 0.04	1.04 ± 0.02
**DPPG**	16:0	1.25 ± 0.10	1.98 ± 0.32	1.66 ± 0.33
**POPG**	16:0, 18:1	1.12 ± 0.06	1.30 ± 0.21	1.74 ± 0.46
**SOPE (cis)**	18:0, 18:1	1.16 ± 0.19	1.60 ± 0.19	1.57 ± 0.25
**DOPE (cis)**	18:1	1.23 ± 0.02	1.34 ± 0.10	1.71 ± 0.31
**DOPE (trans)**	18:1	1.57 ± 0.14	3.29 ± 0.82	5.28 ± 1.82

Reported are the average and s.e.m. (n = 3).

Supplementary References

1 The Pymol Molecular Graphics System, Version 1.8 (2015).

2 Cong, X. *et al.* Determining Membrane Protein-Lipid Binding Thermodynamics Using Native Mass Spectrometry. *J Am Chem Soc* **138**, 4346-4349, doi:10.1021/jacs.6b01771 (2016).
